# A controlling nutritional status score is an independent predictor for patients with newly diagnosed nasal‐type extranodal NK/T‐cell lymphoma based on asparaginase‐containing regimens

**DOI:** 10.1002/cam4.5706

**Published:** 2023-03-03

**Authors:** Wanchun Wu, Kexin Ren, Xi Chen, Na Li, Huijie Zhou, Ming Jiang, Youhui Yu, Liqun Zou

**Affiliations:** ^1^ Department of Oncology West China Hospital, Sichuan University Chengdu China

**Keywords:** controlling nutritional status score, extranodal NK‐T‐cell lymphoma, nutrition, prognoses, survival

## Abstract

**Background:**

The controlling nutritional status (CONUT) score is a nutritional index that combines serum albumin, total cholesterol, and lymphocyte counts. The potential value of CONUT score for predicting clinical outcomes in patients with nasal‐type extranodal NK/T‐cell lymphoma (ENKTL) has not been explored.

**Methods:**

This study included 374 ENKTL patients treated with asparaginase‐containing regimens from September 2012 to September 2017. Clinical characteristics, treatment efficacy, prognostic factors, and the predictive value of CONUT score were analyzed.

**Results:**

The complete response (CR) and overall response rate (ORR) were 54.8% and 74.6%, respectively. Patients with CONUT scores <2 had higher CR and ORR compared to patients with scores ≥2 (69.1% vs. 48.9% for CR, *p* = 0.001; 90.0% vs. 74.6% for ORR, *p* < 0.001). The 5‐year overall survival (OS) and progression‐free survival (PFS) rates were 61.9% and 57.3%, respectively. Patients with CONUT scores <2 had better survival outcomes than those with scores ≥2 (5‐year OS, 76.1% vs. 56.0%, *p* < 0.001; 5‐year PFS, 74.4% vs. 50.1%, *p* < 0.001). CONUT score ≥2 was identified as an independent poor prognostic factor for both OS and PFS. A CONUT score ≥2 was also associated with poorer survival outcomes in low‐risk ENKTL patients.

**Conclusion:**

A CONUT score ≥2 is a prognostic marker for poor survival in patients with ENKTL and could be used to stratify risk in low‐risk patients.

## Graphical abstract

The controlling nutritional status (CONUT) score is a prognostic marker for survival in patients with nasal‐type extranodal NK/T‐cell lymphoma. Patients with CONUT scores <2 had higher complete response and overall response rates, as well as better overall and progression‐free survival rates. A CONUT score ≥2 was identified as an independent poor prognostic factor for both overall survival and progression‐free survival and could be used to stratify risk in low‐risk patients.

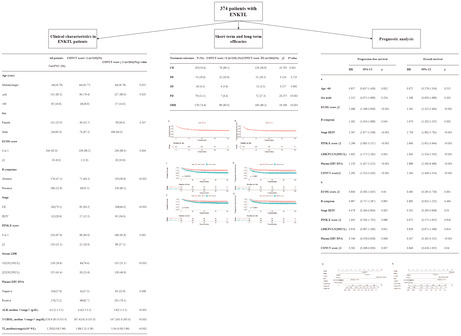

## 1 INTRODUCTION

Extranodal NK/T‐cell lymphoma (ENKTL) is an almost entirely extranodal non‐Hodgkin lymphoma with incidence rates relatively higher rate in South American and Asian regions than in Europe.^1^, ^2^ ENKTL development is linked to infection with Epstein–Barr virus (EBV).^3^ Up to 80% of ENKTL patients have upper aerodigestive tract involvement, and the primary site of involvement is most typically the nasal cavity.^3^, ^4^ The development of asparaginase has led to the marked improvement in the survival outcomes of patients with ENKTL. Nevertheless, a large proportion of patients inevitably relapse and become refractory, and prognosis is deemed unsatisfactory.^5^, ^6^, ^7^

In the anthracycline‐based era, several prognostic models for ENKTL have been developed, such as the International Prognostic Index (IPI) and the Korean Prognostic Index (KPI). However, based on these models, the survival of patients with ENKTL could not be accurately stratified in the nonanthracycline era.^8^, ^9^ Therefore, some new prognostic models were proposed for ENKTL patients. The prognostic model for natural killer lymphoma with Epstein–Barr virus (PINK‐E) has been recommended for predicting the prognosis of ENKTL patients by NCCN guidelines, but it has proven ineffective in stratifying early‐stage patients.^10^ The nomogram‐revised risk index (NRI) showed a better stratification for ENKTL patients than other models (IPI, KPI, and PINK‐E), especially for early‐stage patients.^11^ However, patients' comorbidities have not been considered in these indexes.

In cancer patients, malnutrition is a common comorbidity and is strongly correlated with both treatment efficacy and patient survival.^12^, ^13^ As an efficient index for the early detection and ongoing screening of malnutrition, the controlling nutritional status (CONUT) score, originally reported in 2005, consists of total lymphocyte counts (TL), total cholesterol level (T‐CHOL), and serum albumin (ALB).^14^ Currently, several studies have shown that the CONUT score has prognostic value for hematological malignancies, such as diffuse large B‐cell lymphoma (DLBCL), peripheral T‐cell lymphoma (PTCL), and multiple myeloma (MM).^15^, ^16^, ^17^ However, there have been no studies exploring its prognostic value in patients with ENKTL. In this study, we evaluated the predictive value of the CONUT score toward the prognosis of ENKTL patients receiving asparaginase‐containing regimens.

## 2 METHOD

### 2.1 Patients

We reviewed 374 patients who were newly diagnosed with ENKTL at the West China Hospital of Sichuan University over a 5‐year period (September 2012–2017). Criteria for inclusion were: (a) diagnosed with ENKTL by immunohistochemistry according to the WHO classification criteria established in 2008^18^; (b) 18 years of age or older; (c) chemotherapy regimens including L‐asparaginase or pegaspargase; (d) absence of infection or other malignancies; and (e) availability of complete clinical data. Ethical approval was sought and granted for this retrospective study by the research ethics committee of the West China Hospital (ID: SCHX‐2022‐64).

Pretreatment clinical and laboratory data (collected within 7 days prior to treatment) were compiled for analysis, including Eastern Cooperative Oncology Group performance status (ECOG) score, B symptoms, lactate dehydrogenase (LDH), peripheral plasma EBV‐DNA, TL, T‐CHOL, and ALB. Disease staging and efficacy assessment were administered through positron emission tomography/computed tomography. Additionally, PINK‐E scores were computed for analysis.

### 2.2 CONUT score

Data analysis included the calculation of CONUT scores.^14^ Sub‐scores were assigned according to specific parameters, and the CONUT score was calculated as the sum of these sub‐scores. CONUT parameters and subs‐scores included (a) TL, ≥1.60, 1.20–1.59, 0.80–1.19, <0.80 × 10^9^/L scored as 0, 1, 2, and 3 points, respectively; (b) T‐CHOL, ≥180, 140–179, 100–139, and <100 mg/dL scored as 0, 1, 2, and 3 points, respectively; (c) ALB, ≥3.50, 3.00–3.49, 2.50–2.99, and <2.50 g/dL scored as 0, 2, 4, and 6 points, respectively. Furthermore, following the classification method employed in the previous study, these scores were used to divide patients into two groups: a normal nutritional status group (CONUT score <2) and an undernutrition group (CONUT score ≥2), and the differences in clinical outcomes for these two groups were assessed.^14^

### 2.3 Treatment

All 374 patients received chemotherapy (*n* = 105) or chemoradiotherapy (269). Chemotherapy treatments were based on L‐asparaginase or pegaspargase and were mainly comprised of the following regimens: L‐asparaginase, cisplatin, etoposide, and dexamethasone; L‐asparaginase, vincristine, and prednisone; gemcitabine, L‐asparaginase, ifosfamide, dexamethasone, and etoposide; or pegaspargase, gemcitabine, and oxaliplatin. The aforementioned chemotherapy regimens were administered based on previous studies.^19^, ^20^, ^21^, ^22^ The patients' median chemotherapy cycle was 4 (range, 2–8 cycles). Two hundred eighty‐one patients were treated with a median radiotherapy dose of 50 Gy (range 44–60 Gy) at the involved field, with each fraction of 1.8–2.0 Gy, totaling 5 fractions per week.

At initial diagnosis with early‐stage ENKTL, patients received chemotherapy combined with involved‐field radiation therapy. At the initial diagnosis with advanced stage ENKTL, patients received consolidation radiation therapy of the primary tumor site or local residual lesion after completing planned chemotherapy. In addition, patients could elect to receive hematopoietic stem cell transplantation (HSCT) after achieving complete response (CR) or partial response (PR). Due to a lack of consensus, treatment methods varied and depended largely on physician choice.

### 2.4 Efficacy evaluation

Treatment efficacy evaluation was carried out post‐therapy. Responses were classified based on the response criteria of malignant lymphoma as: CR, PR, stable disease (SD), and progressive disease (PD).^23^ The overall response rate (ORR) was calculated as the percentage of patients achieving either CR or PR. Overall survival (OS) was designated as the time interval between patient diagnosis and the last recorded follow‐up or death from any cause. Similarly, progression‐free survival (PFS) was used to define the period of time between initial diagnosis and disease recurrence, disease progression, the last recorded follow‐up, or death.

### 2.5 Statistical analysis

Continuous variables are presented as median values and ranges. Categorical variables are displayed in the form of frequencies with percentages, and correlations between them were identified via the chi‐square test. To compare these groups, the Mann–Whitney *U*‐test was applied. The Kaplan–Meier method was used to analyze survival outcome data, and the log‐rank test was performed for the comparison of the survival curves. Independent prognostic factors related to ENKTL were identified via Cox regression analyses. These analyses served as the basis for establishing prognostic nomograms for PFS and OS. Two‐sided *p* < 0.05 were considered statistically significant. SPSS version 22.0 (SPSS Inc.) was used for statistical analyses. R (version 4.2.1) was used for prognostic nomograms.

## 3 RESULTS

### 3.1 Patient characteristics

In this study, 374 patients were enrolled for data analysis (Table 1). The median age was 44 years (range, 18–79 years), and 63 (16.8%) patients were older than 60 years. Approximately two‐thirds (65.0%) of the patients were male. Approximately 198 (52.9%) patients had B symptoms. Patients with stage I/II disease numbered 262 (70.1%). Most patients (92.0%) had ECOG scores of 0 or 1, and 155 (41.4%) patients had elevated LDH levels. Two hundred eighty‐five patients (72.2%) had plasma‐positive EBV‐DNA, which was defined as any detectable EBV‐DNA. A total of 254 (67.9%) patients were considered low‐risk based on PINK‐E scoring. The median ALB was 4.0 g/dL, the median T‐CHOL was 158.9 mg/dL, and the median TL was 1.292 x 10^9^/L.

**TABLE 1:** Correlation between the controlling nutritional status score and clinical characteristics in ENKTL patients

	All patients (*n* = 374) (%)	CONUT score <2 (*n* = 110) (%)	CONUT score ≥2 (*n* = 264) (%)	*p‐*Value
Age (years)				
Median(range)	44(18–79)	44(18–77)	44(18–79)	0.051
≤60	311 (83.2)	84 (76.4)	227 (86.0)	0.023
>60	63 (16.8)	26(26.6)	37 (14.0)	
Sex				
Female	131 (35.0)	36 (32.7)	95(36.0)	0.547
Male	243(65.0)	74 (67.3)	169 (64.0)	
ECOG score				
0 or 1	344 (92.0)	108 (98.2)	236 (89.4)	0.004
≥2	30 (8.0)	2 (1.8)	28 (10.6)	
B symptoms				
Absence	176 (47.1)	71 (64.5)	105(39.8)	<0.001
Presence	198 (52.9)	39(35.5)	159 (60.2)	
Stage				
I/II	262(70.1)	93 (84.5)	169(64.0)	<0.001
III/IV	112(29.9)	17 (15.5)	95 (36.0)	
PINK‐E score				
0 or 1	254 (67.9)	88 (80.0)	166 (62.9)	0.001
≥2	120 (32.1)	22 (20.0)	98 (37.1)	
Serum LDH				
<ULN(250 U/L)	219 (58.6)	84(76.4)	135 (51.1)	<0.001
≥ULN(250 U/L)	155 (41.4)	26 (23.6)	129 (48.9)	
Plasma EBV DNA				
Negative	104(27.8)	41(37.3)	63 (23.9)	0.008
Positive	270(72.2)	69(62.7)	201 (76.1)	
ALB, median (range) (g/dL)	4.0 (2.1–5.5)	4.4(3.5–5.2)	3.9(2.1–5.5)	<0.001
T‐CHOL, median (range) (mg/dL)	158.9 (61.0–351.0)	187.4(142.0–351.0)	147.1(61.0–265.0)	<0.001
TL, median (range) (×10^9^/L)	1.292(0.08–5.96)	1.89(1.21–3.39)	1.04 (0.08–5.96)	<0.001

Abbreviations: ALB, serum albumin; CONUT, controlling nutritional status; EBV, Epstein–Barr virus; ECOG score, Eastern Cooperative Oncology Group performance status; LDH, lactate dehydrogenase; PINK‐E, prognostic index of natural killer lymphoma with Epstein–Barr virus; T‐CHOL, total cholesterol level; TL, total lymphocyte count.

### 3.2 Relationships between CONUT score and clinical variables

Nutritional status was normal (<2) and undernutrition (≥2) in 110 (29.4%) and 264 (70.6%) patients, respectively. The correlations identified between the CONUT score and clinical variables are listed in Table 1. There were important associations between age (*p* = 0.023), ECOG score (*p* = 0.004), B symptoms (*p* < 0.001), stage (*p* < 0.001), PINK‐E score (*p* = 0.001), serum LDH (*p* < 0.001), plasma EBV‐DNA (*p* = 0.008), and CONUT score. However, no correlation was found between CONUT score and sex among these patients (*p* = 0.547).

### 3.3 The treatment response of asparaginase‐based regimens

The short‐term efficacy of 374 patients was assessed. Asparaginase‐based regimens achieved promising treatment responses, including 205 (54.8%) CRs, 74 (19.8%) PRs, 16 (4.3%) SDs, and 79 (21.1%) PDs, with an ORR of 74.6%. Among the CONUT score <2 group, there were 76 (69.1%) CRs, 23 (20.9%) PRs, 4 (3.6%) SDs, and 7 (6.4%) PDs. There were 129 (48.9%) CRs, 51 (19.3%) PRs, 12 (4.5%) SDs, and 72 (27.3%) PDs in the CONUT score ≥2 group. The CR and ORR of the CONUT score <2 group were clearly higher than those of the CONUT score ≥2 group (CR, 69.1% vs. 48.9%, *p* = 0.001; ORR, 90.0% vs. 74.6%, *p* < 0.001). Furthermore, it was found that the PD of the CONUT score ≥2 group was consistently higher when directly compared with the PD of the CONUT score <2 group (27.3% vs. 6.4%, *p* < 0.001). However, no differences could be found in PR (*p* = 0.725) or SD (*p* = 0.692) between the two groups (Table S1).

### 3.4 The long‐term efficacy of asparaginase‐based regimens

**FIGURE 1:**
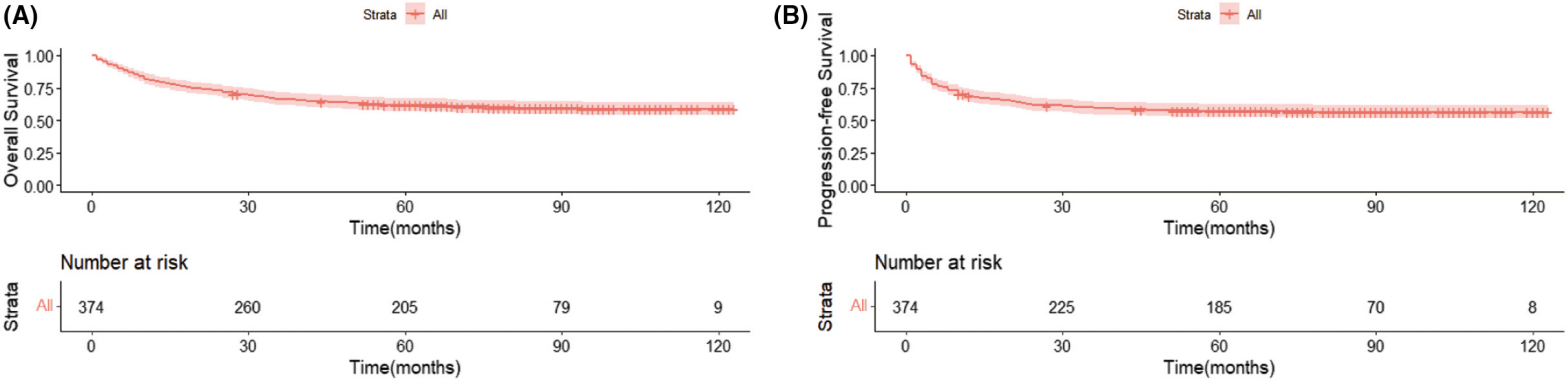
Overall survival (A) and progression‐free survival (B) of asparaginase‐based chemotherapy for extranodal NK‐T‐cell lymphoma patients. ENKTL, extranodal NK‐T‐cell lymphoma; OS, overall survival; PFS, progression‐free survival.

**FIGURE 2:**
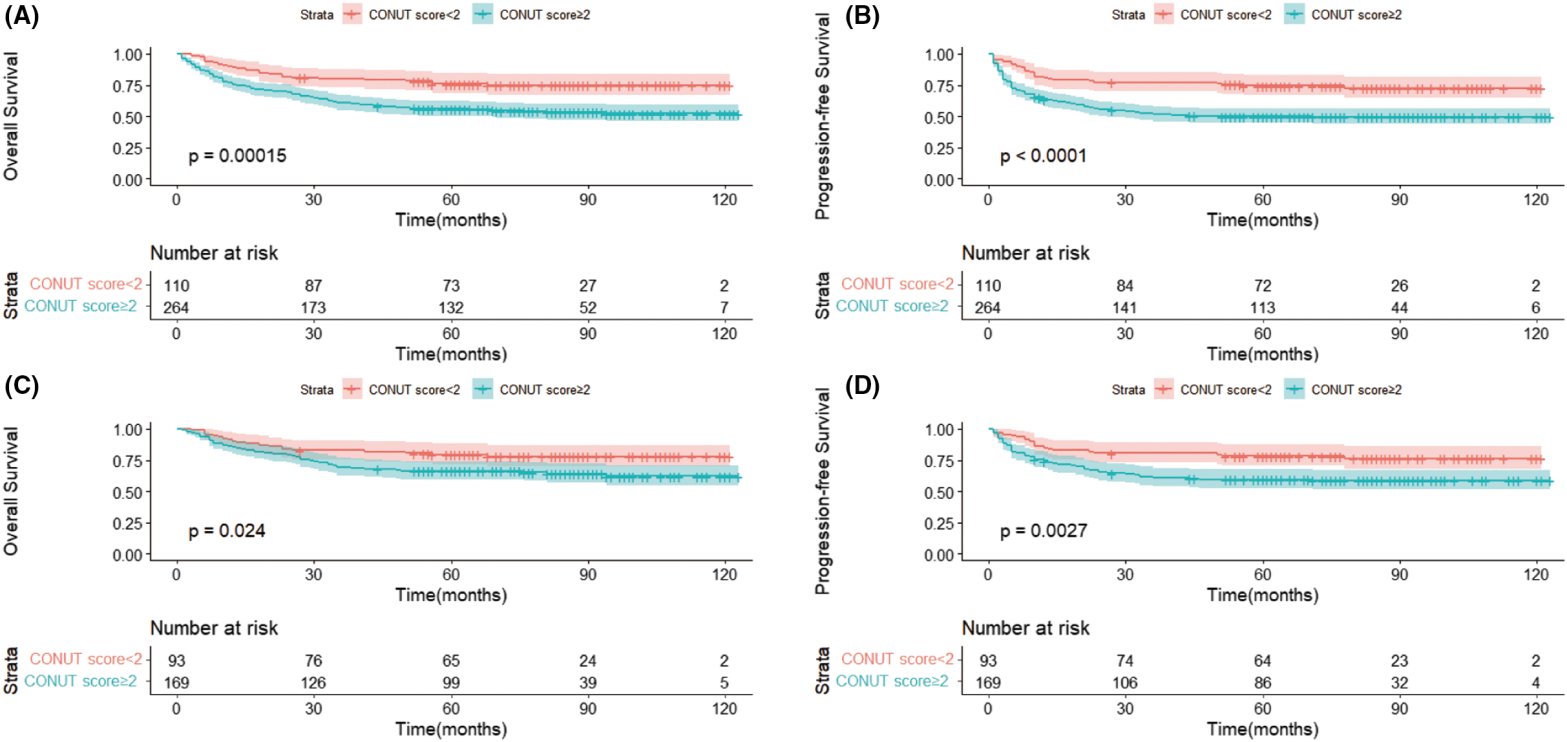
Overall survival (OS) (A) and progression‐free survival (PFS) (B) of controlling nutritional status (CONUT) score <2 and ≥2 groups in extranodal NK‐T‐cell lymphoma (ENKTL) patients; the OS (C) and PFS (D) of CONUT score <2 and ≥2 groups in early‐stage ENKTL patients. ENKTL, extranodal NK‐T‐cell lymphoma; CONUT, controlling nutritional status; OS, overall survival; PFS, progression‐free survival.

The median follow‐up of patients was 82 months (95% CI, 78.3–85.7 months). After the last follow‐up, a total of 161 PFS events were recorded. During the initial therapy, 79 patients experienced PD. After the initial therapy, 69 patients experienced disease recurrence. At the last follow‐up, 25 patients were still disease free following the first relapse or progression. Of them, seven patients underwent HSCT (auto‐HSCT, *n* = 5; allo‐HSCT, *n* = 2) after receiving SMILE (dexamethasone, methotrexate, ifosfamide, L‐asparaginase, and etoposide) chemotherapy. Among the 374 patients, 149 died: 136 deaths were attributed to PD, and the other 13 patients died of unreported causes. For all patients, the 5‐year OS and PFS were 61.9% and 57.3%, respectively (Figure 1). Compared with patients at an advanced stage, early‐stage patients had a relatively better OS and PFS (5‐year OS: 70.9% vs. 40.8%, χ^2^ = 41.043, *p* < 0.001; 5‐year PFS: 66.2% vs. 36.3%, χ^2^ = 38.862, *p* < 0.001) (Figure S1). In addition, patients with negative EBV‐DNA had better survival outcomes than those with positive EBV‐DNA (5‐year OS: 84.5% vs. 53.3%, χ^2^ = 32.573, *p* < 0.001; 5‐year PFS: 74.0% vs. 50.8%, χ^2^ = 15.048, *p* < 0.001) (Figure S2). Patients with CONUT scores <2 had better OS and PFS than those with CONUT scores ≥2 (5‐year OS: 76.1% vs. 56.0%, χ^2^ = 14.339, *p* < 0.001; 5‐year PFS: 74.4% vs. 50.1%, χ^2^ = 17.865, *p* < 0.001) (Figure 2A,B). Similarly, early‐stage patients with CONUT scores <2 had better OS and PFS than those with CONUT scores ≥2 (5‐year OS: 79.4% vs. 66.3%, χ^2^ = 5.103, *p* = 0.024; 5‐year PFS: 78.5% vs. 59.5%, χ^2^ = 8.983, *p* = 0.003) (Figure 2C,D).

### 3.5 Prognostic factors

To identify potential relationships between clinical variables and survival for this cohort of ENKTL patients, univariate and multivariate Cox regression analyses were conducted. In univariate Cox regression analyses, we found that an ECOG score ≥2, B symptoms, stage III/IV, PINK‐E score ≥2, LDH ≥250 U/L, positive EBV‐DNA, and CONUT score ≥2 were strongly correlated with PFS and OS (Table 2A). For the multivariate Cox regression analyses, the above clinical variables were included (*p* < 0.05). Through our analysis, it was found that four factors, including ECOG score ≥2, stage III/IV, positive EBV‐DNA, and CONUT score ≥2, were independent predictors for both PFS and OS (Table 2B).

**TABLE 2:** Univariate (A) and multivariate (B) analyses of progression‐free survival and overall survival in ENKTL patients

	Progression‐free survival			Overall survival		
	HR	95% CI	*p‐*Value	HR	95% CI	*p‐*Value
**A**						
Age > 60	0.957	(0.637–1.438)	0.832	0.872	(0.578–1.316)	0.515
Sex male	1.212	(0.871–1.686)	0.254	1.198	(0.850–1.689)	0.303
ECOG score ≥2	2.486	(1.569–3.938)	<0.001	3.561	(2.312–5.484)	<0.001
B symptom	1.381	(1.010–1.888)	0.043	1.676	(1.202–2.335)	0.002
Stage III/IV	2.567	(1.877–3.509)	<0.001	2.738	(1.982–3.781)	<0.001
PINK‐E score ≥2	2.296	(1.681–3.137)	<0.001	2.640	(1.912–3.644)	<0.001
LDH ≥ ULN(250 U/L)	1.602	(1.175–2.183)	0.003	1.843	(1.334–2.545)	<0.001
Plasma EBV DNA	2.150	(1.437–3.215)	<0.001	3.989	(2.300–6.468)	<0.001
CONUT score ≥2	2.293	(1.533–3.430)	<0.001	2.184	(1.439–3.314)	<0.001
**B**						
ECOG score ≥2	0.640	(0.392–1.045)	0.04	0.463	(0.291–0.738)	0.001
B symptom	0.997	(0.717–1.387)	0.985	0.883	(0.623–1.252)	0.484
Stage III/IV	0.478	(0.284–0.804)	0.005	0.501	(0.295–0.849)	0.01
PINK‐E score ≥2	1.005	(0.593–1.702)	0.986	0.972	(0.571–1.655)	0.918
LDH ≥ ULN(250 U/L)	0.918	(0.697–1.380)	0.911	0.958	(0.671–1.368)	0.814
Plasma EBV DNA	0.548	(0.358–0.838)	0.006	0.307	(0.181–0.521)	<0.001
CONUT score ≥2	0.562	(0.369–0.856)	0.007	0.648	(0.418–1.005)	0.04

Abbreviations: CI, confidence interval; CONUT, controlling nutritional status; EBV, Epstein–Barr virus; ECOG, Eastern Cooperative Oncology Group performance status; HR, hazard rate; LDH, lactate dehydrogenase; PINK‐E, prognostic index of natural killer lymphoma with Epstein–Barr virus.

### 3.6 Further stratification through CONUT scores in addition to PINK‐E

**FIGURE 3:**
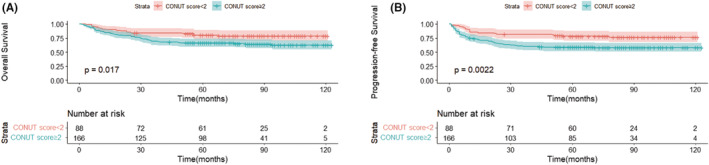
Overall survival (A) and progression‐free survival (B) in low‐risk patients by prognostic index of natural killer lymphoma with Epstein–Barr virus according to controlling nutritional status score. ENKTL, extranodal NK‐T‐cell lymphoma; CONUT, controlling nutritional status; PINK‐E, prognostic index of natural killer lymphoma with Epstein–Barr virus; OS, overall survival; PFS, progression‐free survival.

After multivariate Cox regression analysis, we found that a CONUT score ≥2 was considered a poor factor for PFS and OS; thus, we tried to further stratify ENKTL patients by adding the CONUT score to the PINK‐E prognostic model. Based on the PINK‐E prognostic model, patients were stratified as low‐risk (254 patients), intermediate‐risk (82 patients), or high‐risk (38 patients). Among the intermediate‐risk group and high‐risk group, we did not identify any significant disparity in OS and PFS between the CONUT score <2 and CONUT score ≥2 groups (intermediate‐risk group: OS, χ^2^ = 3.292, *p* = 0.07; PFS, χ^2^ = 3.823, *p* = 0.051; high‐risk group: OS, χ^2^ = 0.366, *p* = 0.545; PFS, χ^2^ = 0.076, *p* = 0.782) (Figure S3). By contrast, among patients with low risk, the OS and PFS was found to be significantly lower for patients with CONUT scores ≥2 (OS, χ^2^ = 5.720, *p* = 0.017; PFS, χ^2^ = 9.393, *p* = 0.002) (Figure 3).

### 3.7 Construction of a nomogram for the prediction of PFS and OS in ENKTL patients

**FIGURE 4:**
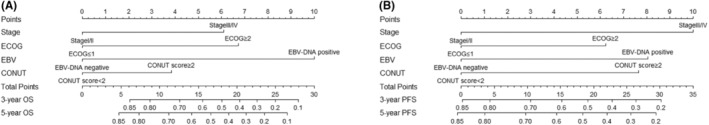
Nomogram for predicting overall survival (A) and progression‐free survival (B). CONUT, controlling nutritional status; EBV, Epstein–Barr virus; ECOG, Eastern Cooperative Oncology Group performance status; OS, overall survival; PFS, progression‐free survival.

Four prognostic indicators potentially affecting the PFS and OS of ENKTL patients were found after multivariate Cox regression analysis, including ECOG score ≥2, stage III/IV, positive EBV‐DNA, and CONUT score ≥2. The nomogram was effective in predicting, from multivariate analysis data, the probabilities of 3‐ and 5‐year PFS and OS with a C‐index of 0.69 and 0.71, respectively (Figure 4).

## 4 DISCUSSION

Through this large‐sample, real‐world study, we demonstrated that the upfront treatment of asparaginase‐based regimens for ENKTL patients achieved promising results in both short‐ and long‐term efficacy. Additionally, a CONUT score ≥2 was found to be a poor independent prognostic factor for ENKTL patients, and it could be utilized to distinguish low‐risk patients based on PINK‐E with inferior OS. Furthermore, based on the result from the multivariate analysis, a prognostic nomogram was constructed to predict 3‐ and 5‐year OS and PFS probability, including ECOG score ≥2, stage III/IV, positive EBV‐DNA, and CONUT score ≥2 as independent factors.

Treatment of ENKTL has evolved, and survival outcomes have improved significantly following the development asparaginase‐based therapy regimens. However, there are only a few retrospective studies discussing the benefits of asparaginase‐containing regimens with short follow‐up. Our large‐scale, real‐world study with a longer median follow‐up (82 months) presents data that support that treatment with asparaginase‐based regimens for ENKTL patients can achieve promising results in both short‐ and long‐term efficacy. The ORR was 74.6%, and 54.8% of patients had CR. The 5‐year OS and PFS rates were 61.9% and 57.3%, respectively. These findings further corroborate the results of previous studies. A multicenter, retrospective analysis of nonanthracycline‐based regimens for 527 ENKTL patients was conducted by Kim et al., and they found that the 3‐year OS and PFS were 59% and 48%, respectively.^10^ Yamaguchi et al.'s study reported the survival outcomes for 358 patients with ENKTL treated with nonanthracycline‐based regimens, and the 5‐year OS and PFS were 56% and 45%, respectively.^24^ In another large‐scale, multicenter study including 166 patients with ENKTL, Fox reported a 5‐year OS of 50% and a 5‐year PFS of 42%.^3^ Therefore, our results provide real‐world evidence supporting the efficacy of asparaginase‐based protocols as initial treatments for patients with ENKTL.

In cancer patients, malnutrition is a common comorbidity and affects disease progression and survival.^25^ Currently, a number of nutritional indexes have been found to be effective prognostic indicators for various cancers, such as the CONUT score and prognostic nutrition index (PNI). In the literature, the PNI has been widely associated with prognosis in ENKTL patients.^26^, ^27^ However, based on asparaginase‐based chemotherapy, there are no published data describing the correlation between the CONUT score and patient prognosis with ENKTL. To our best knowledge, this study is both the first and largest‐scale retrospective study centered on CONUT scores of ENKTL patients.

Through our statistical analysis, we found that the CONUT score was associated with several clinical parameters, including age, ECOG score, B symptoms, stage, PINK‐E score, serum LDH, and plasma EBV‐DNA. This result was similar to previous studies. A study including 99 newly diagnosed patients with PTCL presented strong evidence identifying a significant correlation between higher CONUT scores and worse clinical parameters, including B symptoms, ECOG score ≥2, and high IPI score.^16^ Moreover, a recent retrospective study of 476 DLBCL cases found that a high CONUT score was correlated with older age, advanced stage, abnormal LDH levels, B symptoms, and high NCCN‐IPI score.^15^ Based on the function of ALB, T‐CHOL, and TL, the associations between CONUT score and the basic variables of ENKTL might be clarified, as ALB, for an example, is used for the assessment of nutritional status as well as immune function.^28^ Most cancer patients diagnosed with hypoalbuminemia were placed in the advanced stage category. When the ALB level is less than 30 g/L, the body might not be able to resist the invasion of foreign pathogens, resulting in the release of inflammatory cytokines, which might cause B symptoms. T‐CHOL has important function in the formation of lipid microdomains in cancer cells, which controls cancer cell signaling.^29^ Hypocholesterolemia is associated with worse clinical characteristics (high IPI score, advanced stage, B symptoms, and elevated LDH) in patients with DLBCL.^30^ Lymphocytes are an important part of the patient's immune system and can inhibit tumor cell proliferation and metastasis, induce apoptosis, and play a key role in immune surveillance.^31^ An increasing number of studies have demonstrated an association between low TL at diagnosis and more adverse clinical features.^32^

Furthermore, our analysis suggested that a CONUT score ≥2 was a poor independent prognostic indicator for both OS and PFS. This result was compatible with previous reports. For instance, in a study that stipulated a higher cutoff value at CONUT score ≥4 in analyzing patients with symptomatic MM, a high CONUT score was independently linked to worse OS when compared to patients in the CONUT score <4 group (median OS, 64.1 months vs. not reached; *p* = 0.011).^17^ Moreover, Nagata et al. reported that a high CONUT score was an independent prognostic marker for the survival outcomes of DLBCL patients.^15^ In their study, 476 patients were included, and the 5‐year OS was 83.2% in patients with low CONUT scores compared to 49% in those with high CONUT scores. Our study included 374 patients with ENKTL who underwent treatment with an asparaginase‐based regimen, and the CONUT score <2 group exhibited better 5‐year OS and PFS (5‐year OS, 76.1% vs. 56.0%, *p* = 0.001; 5‐year PFS, 74.4% vs. 50.1%, *p* < 0.001) than the high CONUT score ≥2 group. Hence, it might be necessary to establish new treatments or individualized treatments for patients with a high CONUT score in the future.

While a greater proportion of patients are considered low‐risk according to PINK‐E, a significant difference in OS was not observed between the low‐risk and intermediate‐risk groups (*p* = 0.068).^10^ In our study, we showed that approximately 70% of ENKTL patients were low‐risk according to PINK‐E, but PINK‐E was not an independent predictor for OS. However, the combination of the PINK‐E prognostic system with the CONUT score can serve to refine low‐risk patients. Through stratified analysis, a CONUT score ≥2 was found to be an adverse predictor for OS in low‐risk ENKTL patients (χ^2^ = 5.720, *p* = 0.017). Inspired by the results of this study, nutritional interventions might be useful to further improve the survival outcomes of low‐risk ENKTL patients with CONUT scores ≥2.

In addition, in our study, we developed a predictive nomogram for the prognosis of ENKTL patients based on the results of multivariate Cox regression analyses. The factors incorporated into this nomogram for PFS and OS included ECOG score ≥2, stage III/IV, positive EBV‐DNA, and CONUT score ≥2. The C‐index demonstrated that the nomogram for OS developed in this study has good discrimination.

Although this is the first study to evaluate the prognostic value of the CONUT score in ENKTL patients, certain limitations should be considered. First, the data included in this study were taken from only a single center and included no verification group. Moreover, treatment methods varied, and this potentially induced a survival bias in the data. Therefore, a multicenter prospective study should be designed to further assess and evaluate the role of the CONUT score in prognosis for ENKTL patients.

## 5 CONCLUSION

In summary, a significant correlation was found between a CONUT score ≥2 and inferior survival outcomes as well as worse therapeutic response in patients with ENKTL, and it could further stratify OS in low‐risk patients. Considering the results of our study, the CONUT score could potentially be applicable as a prognostic predictor for ENKTL patients.

## AUTHOR CONTRIBUTIONS

**Wanchun Wu:** Conceptualization (lead); data curation (equal); formal analysis (equal); writing – original draft (lead); writing – review and editing (equal). **Kexin Ren:** Data curation (equal); formal analysis (equal); methodology (equal); writing – review and editing (equal). **Xi Chen:** Data curation (equal); visualization (equal); writing – review and editing (equal). **Na Li:** Data curation (equal); writing – review and editing (equal). **Huijie Zhou:** Writing – review and editing (equal). **Ming Jiang:** Conceptualization (equal); writing – review and editing (equal). **Youhui Yu:** Writing – review and editing (equal). **Liqun Zou:** Conceptualization (equal); supervision (equal); writing – review and editing (equal).

## FUNDING INFORMATION

This work received no specific grant from any funding agency in the public, commercial, or not‐for‐profit sectors.

## CONFLICT OF INTEREST STATEMENT

None.

## Supporting information


Figure S1.
Figure S2.
Figure S3.
Table S1.

## Data Availability

Data sharing is not applicable to this article as no new data were created or analyzed in this study.
