# Supplementary material for: A controlling nutritional status score is an independent predictor for patients with newly diagnosed nasal‐type extranodal NK/T‐cell lymphoma based on asparaginase‐containing regimens
Source: Cancer Med. 2023 Mar 3;12(8):9439–48. doi: 10.1002/cam4.5706 (PMC10166885; doi:10.1002/cam4.5706)
Supplement: Supplementary file 1 — Figure S1. Figure S2. Figure S3. Table S1. [file CAM4-12-9439-s001.docx]

**Table S1.** The correlation between CONUT score and treatment outcomes in ENKTL patients

| **Treatment outcomes** | **N (%)** | **CONUT score <2 (n=110) (%)** | **CONUT score ≥2 (n=264)(%)** | **χ2** | ***P* value** |
| --- | --- | --- | --- | --- | --- |
| **CR** | 205(54.8) | 76 (69.1) | 129 (48.9) | 10.791 | 0.001 |
| **PR** | 74 (19.8) | 23 (20.9) | 51 (19.3) | 0.124 | 0.725 |
| **SD** | 16 (4.3) | 4 (3.6) | 12 (4.5) | 0.157 | 0.692 |
| **PD** | 79 (21.1) | 7 (6.4) | 72 (27.3) | 20.375 | <0.001 |
| **ORR** | 279 (74.6) | 99 (90.0) | 180 (68.2) | 19.506 | <0.001 |

Abbreviations: CR, complete remission; CONUT, controlling nutritional status; ORR, overall response rate; PD, progressive disease; PR, partial remission; SD, stable disease.


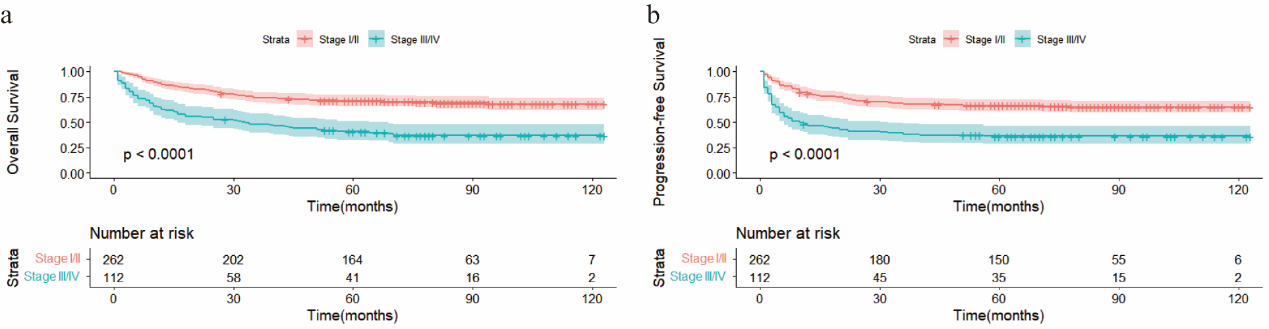


**Figure S1.** The OS (a) and PFS (b) of early-stage and advanced-stage groups of ENKTL patients.

**Abbreviations:** ENKTL, extranodal NK-T-cell lymphoma; OS, overall survival; PFS, progression-free survival.


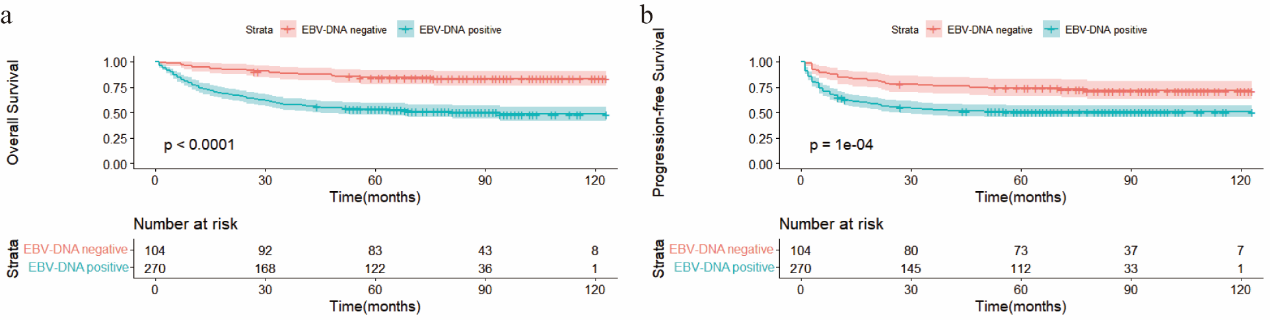


**Figure S2.** The OS (a) and PFS (b) of negative EBV-DNA and positive EBV-DNA groups in ENKTL patients.

Abbreviations: EBV, Epstein‒Barr virus; ENKTL, extranodal NK-T-cell lymphoma; OS, overall survival; PFS, progression-free survival.


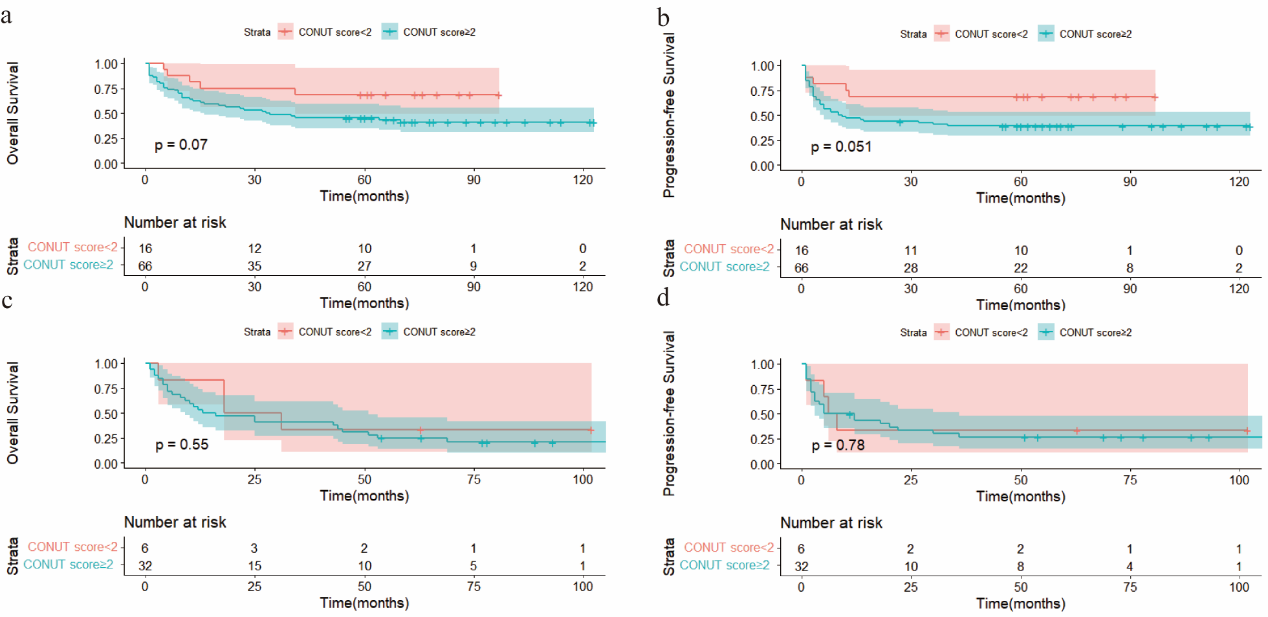


**Figure S3**. The OS and PFS in intermediate- (a, b) and high-risk (c, d) patients by PINK-E according to CONUT score.

Abbreviations: ENKTL, extranodal NK-T-cell lymphoma; CONUT, controlling nutritional status; PINK-E, prognostic index of natural killer lymphoma with Epstein‒Barr virus; OS, overall survival; PFS, progression-free survival.
